# Glyceraldehyde-Derived Pyridinium Evokes Renal Tubular Cell Damage via RAGE Interaction

**DOI:** 10.3390/ijms21072604

**Published:** 2020-04-09

**Authors:** Ami Sotokawauchi, Nobutaka Nakamura, Takanori Matsui, Yuichiro Higashimoto, Sho-ichi Yamagishi

**Affiliations:** 1 Department of Pathophysiology and Therapeutics of Diabetic Vascular Complications, Kurume University School of Medicine, Kurume 830-0011, Japan; sotokawauchi_ami@med.kurume-u.ac.jp (A.S.); k9389438@kadai.jp (N.N.); matsui_takanori@med.kurume-u.ac.jp (T.M.); 2 Department of Chemistry, Kurume University School of Medicine, Kurume 830-0011, Japan; higashiy@med.kurume-u.ac.jp; 3 Division of Diabetes, Metabolism, and Endocrinology, Department of Medicine, Showa University School of Medicine, Tokyo 142-8666, Japan

**Keywords:** GLAP, RAGE, diabetic nephropathy, proximal tubular cells

## Abstract

Glyceraldehyde-derived advanced glycation end products (glycer-AGEs) contribute to proximal tubulopathy in diabetes. However, what glycer-AGE structure could evoke tubular cell damage remains unknown. We first examined if deleterious effects of glycer-AGEs on reactive oxygen species (ROS) generation in proximal tubular cells were blocked by DNA-aptamer that could bind to glyceraldehyde-derived pyridinium (GLAP) (GLAP-aptamer), and then investigated whether and how GLAP caused proximal tubular cell injury. GLAP-aptamer and AGE-aptamer raised against glycer-AGEs were prepared using a systemic evolution of ligands by exponential enrichment. The binding affinity of GLAP-aptamer to glycer-AGEs was measured with a bio-layer interferometry. ROS generation was evaluated using fluorescent probes. Gene expression was analyzed by reverse transcription-polymerase chain reaction (RT-PCR). GLAP-aptamer bound to glycer-AGEs with a dissociation constant of 7.7 × 10^−5^ M. GLAP-aptamer, glycer-AGE-aptamer, or antibodies directed against receptor for glycer-AGEs (RAGE) completely prevented glycer-AGE- or GLAP-induced increase in ROS generation, MCP-1, PAI-1, or RAGE gene expression in tubular cells. Our present results suggest that GLAP is one of the structurally distinct glycer-AGEs, which may mediate oxidative stress and inflammatory reactions in glycer-AGE-exposed tubular cells. Blockade of the interaction of GLAP-RAGE by GLAP-aptamer may be a therapeutic target for proximal tubulopathy in diabetic nephropathy.

## 1. Introduction

According to the Diabetes Atlas, 9th edition, 2019, an estimated 463 million people worldwide have diabetes, which is an increasing global health burden [1]. Among various complications, diabetic nephropathy is the most common and leading cause of end-stage renal disease, which is associated with the increased risk of cardiovascular disease and total mortality in both type 1 and type 2 diabetic patients [2]. Although strict blood glucose control and management of hypertension with inhibitors of renin-angiotensin system have been shown to significantly reduce the development and progression of diabetic nephropathy, these therapeutic options are far from satisfactory because a substantial population of diabetic patients still develop renal failure [2]. Therefore, identification of residual risk factors is needed for the management of diabetic nephropathy.

We have previously found that DNA-aptamer raised against glyceraldehyde-derived advanced glycation end products (glycer-AGEs), whose formation and accumulation are enhanced in patients with insulin resistance and/or diabetes [2,3,4], attenuate the progression of renal damage in obese type 2 diabetic mice, thus suggesting that glycer-AGEs may be a novel therapeutic target for diabetic nephropathy [5]. Furthermore, glycer-AGEs have also been shown to evoke oxidative stress generation, inflammatory, and fibrotic reactions in human renal proximal tubular cells via the interaction with receptor for AGEs (RAGE) [6]. These observations suggest that interaction of glycer-AGEs with RAGE may play a role in proximal tubulopathy, a key mover of diabetic nephropathy [7]. However, it remains unclear what structurally distinct glycer-AGEs are involved in diabetic nephropathy. Although modification of proteins by glyceraldehyde could generate a large number of AGEs, including glyceraldehyde-derived pyridinium (GLAP), methylglyoxal-derived hydroimidazolone 1, and argpyrimidine [8,9,10,11], GLAP has been identified as one of the major glycer-AGEs existed in diabetic animals or patients [9,12]. Furthermore, we have found that GLAP at 1-10 μg/mL, whose concentrations are comparable with those of the in vivo-diabetic situation, elicits oxidative stress and inflammatory and thrombogenic reactions in endotheliual cells through the interaction with RAGE [12]. Therefore, we examined here whether deleterious effects of glycer-AGEs on reactive oxygen species (ROS) generation in proximal tubular cells were blocked by DNA-aptamer that could bind to GLAP (GLAP-aptamer), and then investigated whether and how GLAP caused proximal tubular cell damage in vitro.

## 2. Results

**Figure 1:**
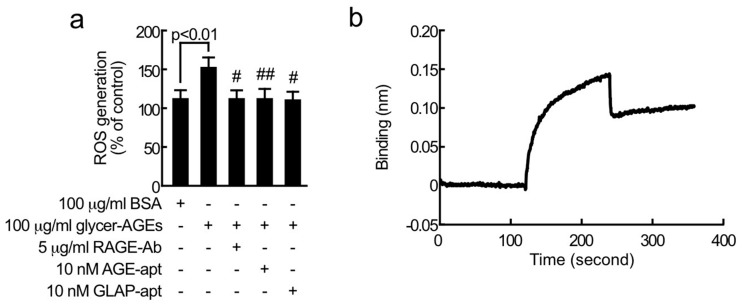
(**a**) Effects of RAGE-Ab, AGE-aptamer (AGE-apt), or GLAP-aptamer (GLAP-apt), on reactive oxygen species (ROS) generation in glycer-AGE-exposed tubular cells. Tubular cells were treated with 100 μg/mL glycer-AGEs or 100 μg/mL non-glycated bovine serum albumin (BSA) in the presence or absence of 5 μg/mL RAGE-Ab, 10 nM AGE-apt, or 10 nM GLAP-apt for 1 h. ROS generation was evaluated by CellRox oxidative stress reagents. *N* = 6–12 per group. # and ##, *p* < 0.05 and *p* < 0.01 compared to the values with 100 μg/mL glycer-AGEs. (**b**) The interaction of GLAP-aptamer to immobilized glycer-AGEs was analyzed by bio-layer interferometry. *N* = 4 per group.

We first examined the effects of GLAP-aptamer on ROS generation in glycer-AGE-exposed proximal tubular cells. As shown in Figure 1a, compared with non-glycated control bovine serum albumin (BSA), glycer-AGEs significantly increased ROS generation in tubular cells, which was completely blocked by 10 nM GLAP-aptamer, 10 nM DNA-aptamer raised against glycer-AGEs (AGE-aptamer), or 5 μg/mL neutralizing rabbit polyclonal antibody directed against RAGE (RAGE-Ab). Bio-layer interferometry analysis revealed that GLAP-aptamer bound to immobilized glycer-AGEs with a dissociation constant (*K*_D_) of 7.7 × 10^−5^ M (Figure 1b).

**Figure 2:**
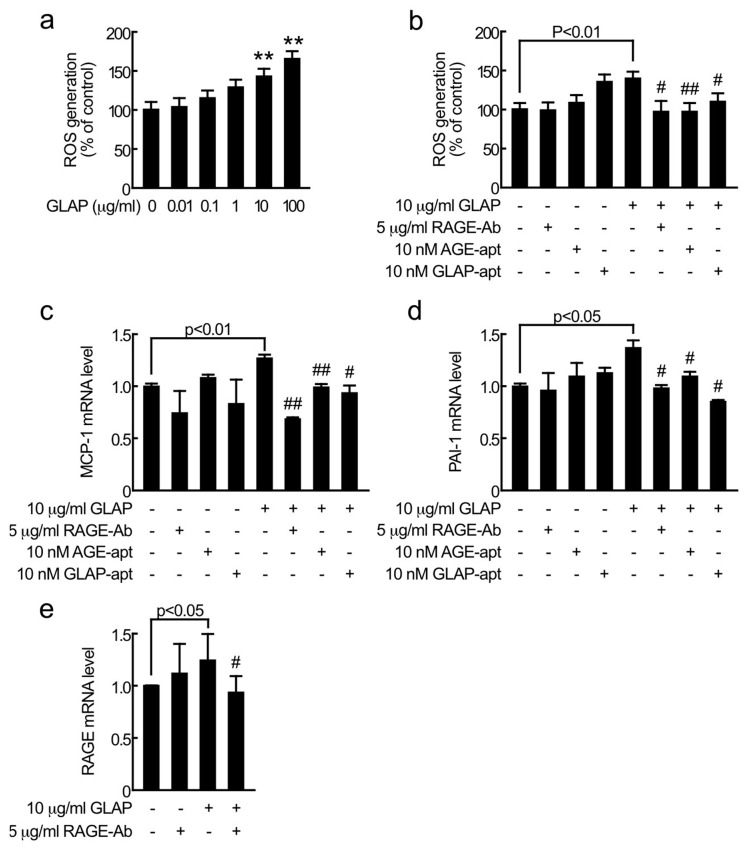
Effects of glyceraldehyde-derived pyridinium (GLAP) or GLAP-aptamer (GLAP-apt) on ROS generation (**a**,**b**), MCP-1 (**c**), PAI-1 (**d**), and RAGE mRNA levels (**e**) in proximal tubular cells. Tubular cells were treated with the indicated concentrations of GLAP in the presence or absence of 5 μg/mL RAGE-Ab, 10 nM AGE-apatmer (AGE-apt), or 10nM GLAP-apt for 1 h (**a**,**b**) or for 4 h (**c**–**e**). ROS generation was evaluated by CellRox oxidative stress reagents. *N* = 6 per group (**c**–**e**). Total RNAs were transcribed and amplified by real-time PCR. Data were normalized by the intensity of 18S rRNA mRNA-derived signals and then related to the control values. (**c**,**d**) *N* = 3 per group. (**e**) *N* = 7 per group. **, *p* < 0.01 compared to the control values. # and ##, *p* < 0.05 and *p* < 0.01 compared to the values with 10 μg/mL GLAP alone, respectively.

We next investigated the effects of GLAP on proximal tubular cells. As shown in Figure 2a, GLAP dose-dependently increased ROS generation in tubular cells; 10 μg/mL and 100 μg/mL GLAP increased the ROS generation by 1.3- and 1.6-fold of control values, respectively. Furthermore, 10 nM GLAP-aptamer, 10 nM AGE-aptamer, or 5 μg/mL RAGE-Ab completely blocked the 10 μg/mL GLAP-induced increase in ROS generation in tubular cells (Figure 2b). While 10 nM AGE-aptamer or 5 μg/mL RAGE-Ab alone did not affect the ROS generation in tubular cells, 10 nM GLAP-aptamer alone modestly increased the ROS generation (Figure 2b).

As shown in Figure 2c–e, 10 μg/mL GLAP significantly increased monocyte chemoattractant protein-1 (MCP-1), plasminogen activator inhibitor-1 (PAI-1), and RAGE mRNA levels in tubular cells, which were completely prevented by the treatment with 10 nM GLAP-aptamer, 10 nM AGE-aptamer or 5 μg/mL RAGE-Ab. Ten nM GLAP-aptamer, 10 nM AGE-aptamer or 5 μg/mL RAGE-Ab alone did not affect gene expressions of MCP-1, PAI-1, or RAGE.

## 3. Discussion

We have previously shown that (1) engagement of RAGE with glycer-AGEs evokes inflammatory, thrombogenic, and fibrotic reactions in human renal proximal tubular cells via ROS generation, (2) sodium-glucose cotransporter 2 (SGLT2)-mediated, high glucose-induced ROS generation augments the glycer-AGE-induced apoptotic cell death of proximal tubular cells via RAGE induction, and (3) inhibitors of SGLT2, such as empagliflozin and tofogliflozin, protect against proximal tubular injury in diabetic animals through its anti-oxidative, anti-inflammatory and anti-fibrotic properties via inhibition of the glycer-AGE-RAGE axis [6,13,14,15,16]. Furthermore, recently, high glucose or AGEs have been shown to promote human renal proximal tubular epithelial cell migration and epithelial-to-mesenchymal transition via oxidative stress generation, all of which were ameliorated by empagliflozin [17]. In addition, an SGLT2 inhibitor, dapagliflozin, inhibited the high glucose-induced inflammatory and fibrotic reactions in human proximal tubular epithelial cells by suppressing the RAGE-downstream signaling pathway [18]. These observations indicate that ROS evoked by glycer-AGE-RAGE interaction in the diabetic kidneys may be a therapeutic target for proximal tubulopathy, a more important prognostic factor than glomerulopathy in terms of renal prognosis in diabetic nephropathy [7].

In this study, we found for the first time that (1) like AGE-aptamer or RAGE-Ab, GLAP-aptamer completely prevented the glycer-AGE-induced increase in ROS generation in proximal tubular cells and (2) GLAP-aptamer bound to glycer-AGEs although its binding affinity was relatively weaker than that of AGE-aptamer (*K*_D_ = 1.4 × 10^−6^ M) [5]. These findings demonstrate that GLAP is actually formed in the process of non-enzymatic glycation of BSA by glyceraldehyde in vitro, thus suggesting that GLAP-aptamer may suppress the ROS generation in glycer-AGE-exposed tubular cells by blocking the GLAP-RAGE interaction.

To further examine the clinical relevance of GLAP in proximal tubular cell damage in diabetic nephropathy, we next investigated whether 10 μg/mL GLAP, which concentration is comparable with that of in vivo-diabetic situations [12], could cause proximal tubular cell injury. As with the case of glycer-AGE-exposed cells, we found that 10 μg/mL GLAP significantly stimulated oxidative stress generation and increased MCP-1 and PAI-1 mRNA levels in proximal tubular cells, all of which were completely blocked by RAGE-Ab, AGE-aptamer, or GLAP-aptamer. These observations indicate that GLAP could elicit oxidative stress generation and inflammatory reactions in proximal tubular cells via the interaction with RAGE. We have previously shown that AGE-aptamer binds to GLAP with *K*_D_ of 3.3 × 10^−5^ M and inhibits the GLAP-induced endothelial cell damage [12]. Therefore, AGE-aptamer may exert protective effects against experimental diabetic nephropathy in obese type 2 diabetic mice partly by blocking the interaction of GLAP with RAGE [5,12].

In the present study, RAGE-Ab completely prevented the up-regulation of RAGE mRNA levels in GLAP-exposed tubular cells. Given the facts that MCP-1 and PAI-1 contribute to tubulointerstitial injury and fibrosis in diabetic nephropathy [6,19,20,21], GLAP-RAGE interaction-mediated RAGE gene induction might make a vicious cycle, thereby further potentiating tubular cell damage in diabetic nephropathy. Taken together, our present findings suggest that GLAP is one of the structurally distinct glycer-AGEs, which may mediate oxidative stress generation, inflammatory and fibrotic reactions in glycer-AGE-exposed proximal tubular cells. Since the results of this study were based on experiments performed on a single cell line, an animal model experiment is needed to examine whether GLAP-aptamer may be a novel therapeutic tool for proximal tubulopathy in diabetic nephropathy. Furthermore, although the present findings suggest that among the different structurally identified AGEs, GLAP was a major AGE that could mediate the deleterious effects of glycer-AGEs, it would be interesting to test the effects of other glycer-AGEs, such as methylglyoxal-derived hydroimidazolone 1 and argpyrimidine on proximal tubular cells using the same protocol as the one used for GLAP.

## 4. Materials and Methods

Glyer-AGEs and non-glycated control BSA were prepared as described previously [12]. In brief, BSA was incubated under sterile conditions with or without glyceraldehyde for 7 days. Then, unbounded sugars were removed by dialysis against phosphate-buffered saline. GLAP was synthesized by incubating *N*-acetyl-L-lysine with glyceraldehyde for 7 days [12]. GLAP was synthesized according to the method of Usui et al. [9,12]. In brief, glyceraldehyde (0.2 M) and *N*-acetyl-L-lysine (0.1 M) were dissolved in 0.2 M sodium phosphate buffer (pH 7.4), and incubated at 37 °C. After a week, the reaction mixture was filtered, and then put on a C8 column on preparative reversed phase high-performance liquid chromatography [9]. Sections of AGE-aptamer and GLAP-aptamer were performed using systemic evolution of ligands by exponential enrichment; sequences of AGE-aptamer and GLAP-aptamer were 5′-tgTAgcccgAgTATcATTcTccATcgcccccAgATAcAAg-3′ and 5′- gcGggTtgGgaGccActAgtAgcAacGtgCgaCccTctAcgAagCaaAccAtcCtcA-3′, where 5′ side of phosphorothioate nucleotides are indicated as capital letters [12]. RAGE-Ab, which recognizes the amino acid residues 167-180 of human RAGE, was prepared as described previously [12]. The interaction of GLAP-aptamer to immobilized glycer-AGEs on the biosensor tip surface was analyzed by bio-layer interferometry using a BLItz instrument (ForteBio, Inc., Menlo Park, CA, USA).

Human primary cultured renal proximal tubular epithelial cells were obtained from Lonza Group Ltd. (Basel, Switzerland) and maintained in basal medium containing 0.5% fetal bovine serum according to the supplier’s instructions [12]. Cell experiments were carried out in a serum-free basal medium. Tubular cells were treated with 100 μg/mL glycer-AGEs, 100 μg/mL non-glycated BSA, or the indicated concentrations of GLAP in the presence or absence of 5 μg/mL RAGE-Ab, 10 nM AGE-apatmer, or 10 nM GLAP-aptamer for 1 h (ROS generation assay) or for 4 h (real-time reverse transcription-polymerase chain reaction (RT-PCR) analysis). ROS generation was evaluated by CellRox oxidative stress reagents (Thermo Fisher Scientific, Waltham, MA, USA) according to the manufacturer’s recommendation [22]. Total RNA was extracted with NucleoSpin RNA kit (Takara Bio Inc., Shiga, Japan) according to the manufacturer’s instructions. Quantitative real-time RT-PCR was performed using Assay-on-Demand and TaqMan 5 fluorogenic nuclease chemistry (Life Technologies Japan Ltd., Tokyo, Japan). Gene expressions of MCP-1, PAI-1, and RAGE were evaluated by RT-PCR analyses; IDs of primers for MCP-1, PAI-1, RAGE, and 18S rRNA gene were Hs00234140_m1, Hs01126606_m1, Hs00542592_g1, and Hs9999901_s1, respectively [8].

All values were presented as mean ± standard deviation. One-way ANOVA followed by Dunnett’s test for Figure 2a or student’s t-test for rest of all were performed for statistical comparisons; *p* < 0.05 was considered significant.

## 5. Conclusions

Our present results suggest that GLAP is one of the structurally distinct glycer-AGEs, which may mediate oxidative stress and inflammatory reactions in glycer-AGE-exposed tubular cells. Blockade of the interaction of GLAP-RAGE by GLAP-aptamer may be a therapeutic target for proximal tubulopathy in diabetic nephropathy.
